# Prevalence, indications, and complications of caesarean section in health facilities across Nigeria: a systematic review and meta-analysis

**DOI:** 10.1186/s12978-023-01598-9

**Published:** 2023-06-02

**Authors:** Itohan Osayande, Olakunmi Ogunyemi, Uchenna Gwacham-Anisiobi, Abimbola Olaniran, Sanni Yaya, Aduragbemi Banke-Thomas

**Affiliations:** 1 School of Human Sciences, University of Greenwich, Old Royal Naval College, Park Row, London, SE10 9LS UK; 2 Lagos State Ministry of Health, Ikeja, Lagos Nigeria; 3 Nuffield Department of Population Health, University of Oxford, Oxford, UK; 4 Global Health, KIT Royal Tropical Institute, Amsterdam, Netherlands; 5 School of International Development and Global Studies, Faculty of Social Sciences, University of Ottawa, Ottawa, Canada; 6 The George Institute for Global Health, Imperial College London, London, UK; 7 Faculty of Epidemiology and Population Health, London School of Hygiene and Tropical Medicine, London, UK; 8 Maternal and Reproductive Health Research Collective, Lagos, Nigeria

**Keywords:** Caesarean delivery, Emergency obstetric care, Prevalence, Indication, Complication, Nigeria, Systematic review

## Abstract

**Background:**

Over 80,000 pregnant women died in Nigeria due to pregnancy-related complications in 2020. Evidence shows that if appropriately conducted, caesarean section (CS) reduces the odds of maternal death. In 2015, the World Health Organization (WHO), in a statement, proposed an optimal national prevalence of CS and recommended the use of Robson classification for classifying and determining intra-facility CS rates. We conducted this systematic review and meta-analysis to synthesise evidence on prevalence, indications, and complications of intra-facility CS in Nigeria.

**Methods:**

Four databases (African Journals Online, Directory of Open Access Journals, EBSCOhost, and PubMed) were systematically searched for relevant articles published from 2000 to 2022. Articles were screened following the PRISMA guidelines, and those meeting the study’s inclusion criteria were retained for review. Quality assessment of included studies was conducted using a modified Joanna Briggs Institute’s Critical Appraisal Checklist. Narrative synthesis of CS prevalence, indications, and complications as well as a meta-analysis of CS prevalence using R were conducted.

**Results:**

We retrieved 45 articles, with most (33 (64.4%)) being assessed as high quality. The overall prevalence of CS in facilities across Nigeria was 17.6%. We identified a higher prevalence of emergency CS (75.9%) compared to elective CS (24.3%). We also identified a significantly higher CS prevalence in facilities in the south (25.5%) compared to the north (10.6%). Furthermore, we observed a 10.7% increase in intra-facility CS prevalence following the implementation of the WHO statement. However, none of the studies adopted the Robson classification of CS to determine intra-facility CS rates. In addition, neither hierarchy of care (tertiary or secondary) nor type of facility (public or private) significantly influenced intra-facility CS prevalence. The commonest indications for a CS were previous scar/CS (3.5–33.5%) and pregnancy-related hypertensive disorders (5.5–30.0%), while anaemia (6.4–57.1%) was the most reported complication.

**Conclusion:**

There are disparities in the prevalence, indications, and complications of CS in facilities across the geopolitical zones of Nigeria, suggestive of concurrent overuse and underuse. There is a need for comprehensive solutions to optimise CS provision tailor-made for zones in Nigeria. Furthermore, future research needs to adopt current guidelines to improve comparison of CS rates.

**Supplementary Information:**

The online version contains supplementary material available at 10.1186/s12978-023-01598-9.

## Introduction

Maternal mortality remains a massive challenge for health systems in Africa [1]. As per the most recent estimates published in 2023, one in 42 women in Africa has a lifetime risk of maternal death—the highest across the world. About 70% of the 282,000 global maternal deaths in 2020 occurred in Africa [2]. Nigeria, an African country which is only ranked eighth in the world in terms of fertility rate, contributed the highest number of maternal deaths worldwide (82,000) [2, 3]. As of 2015, the country also had one of the highest perinatal deaths globally and the second-highest stillbirths (313,700) [4].

Evidence shows that access to emergency obstetric care (EmOC), a package of clinical or surgical interventions used to manage potentially life-threatening complications that affect women during pregnancy, childbirth, and the immediate postpartum period, is critical for reducing maternal and perinatal mortality [5]. Caesarean section (CS), one such EmOC intervention, is a form of delivery whereby the mother’s abdomen and uterus are surgically opened to deliver the baby [6, 7]. It serves as an alternative when traditional vaginal delivery could culminate in the death of the mother or the baby [6]. CS is often performed at the recommendation of medical personnel: either during routine pregnancy assessments when scheduled as an elective procedure or as an emergency consideration where the decision is made impromptu because vaginal delivery is deemed too risky [8].

Several factors, which may be absolute or relative clinical indications, can necessitate or predispose a pregnant woman to having a CS. The absolute indications are those situations in which the procedure is necessary to save a life, for example, in the adverse occurrence of uterine rupture [9]. On the other hand, the relative indications, which may not pose an imminent threat to life, can include a previous caesarean delivery, failure to progress with labour following a risk assessment and so on [9]. However, in the absence of any of these indications, it can be performed on request, following an informed maternal decision [10].

Although a CS can help to save the lives of mothers and babies when used appropriately and conducted well, it is also associated with short- and long-term consequences. For example, evidence shows that compared to women who give birth per vagina, those who deliver via a CS have a higher risk of maternal death, more extended hospital stay, uterine rupture in future pregnancy, and peripartum hysterectomy [11]. Furthermore, children born through a CS have a higher risk of neonatal mortality and, on survival, are more prone to developing asthma and childhood obesity [11]. Considering these potential detrimental outcomes, it is important to ensure that this surgical intervention is used adequately and appropriately to prevent adverse outcomes.

Since 1990, the global prevalence of CS has significantly risen from 7 to 21% in 2018 [12, 13]. This increase, which is more pronounced in high-income countries (15 to 35%) compared to low-income countries (< 9%), has raised questions on the ideal CS rate and its associated effects on maternal and child health [14]. Regarding this, the World Health Organization (WHO) released a statement in 2015 highlighting that the optimal prevalence of CS at national level should be approximately 10% because no significant reductions in maternal or child mortality occur beyond this rate [15]. In addition, a CS rate of less than 5% was deemed to indicate an unmet need for CS in Africa [12].

However, though the WHO recognised the importance of monitoring facility-level rates, it did not recommend the ideal CS rate at facility level due to the high heterogeneity of factors (such as case variations in the obstetric population at the facility and clinical management procedures) that influence the intra-facility CS rates [15]. To monitor and compare CS rates within and between health facilities, the WHO recommends the Robson’s classification system as the gold standard to replace the traditional aggregation of CS rates irrespective of the specific population characteristics at the health facility [15]. This system uses specific parameters (pregnancy and previous CS, onset of labour, number of foetuses, foetal lie or presentation and gestational age) to classify pregnant women into ten groups to allow for a uniform assessment of CS rates universally [15].

In Nigeria, there have been several studies that assessed CS rates, indications, and complications. These have either aggregated data from the country’s National Demographic and Health Surveys (NDHS) [16–19] or congregational surveys [20] to assess population-level metrics or collated data at facility level [21–65]. The NDHS provides comprehensive coverage of the population-based CS trends in Nigeria, with the most recent prevalence rate of 2.7% in 2018, which is very low compared to the global guidelines [66]. However, there has not been an attempt to systematically collate the available evidence from facilities across Nigeria to characterise the trends and patterns of CS in the country. To address this gap, we systematically reviewed the literature on prevalence, indications, and complications of CS in Nigerian health facilities, along with a meta-analysis of CS prevalence.

## Methods

### Study design

This systematic review and meta-analysis were conducted following the Preferred Reporting Items for Systematic Reviews and Meta-Analyses (PRISMA) guideline released in 2020 [67]. The protocol was registered on the International Prospective Register of Systematic Reviews (PROSPERO registration number: CRD42022296473).

### Eligibility criteria

Studies were included if they met the following criteria:

- Reported on the prevalence, indications, outcomes and/or complications of CS in health facilities in Nigeria, whether public, private, or religious.
- Conducted between 2000 and 2022 to allow us to capture the trends in CS in Nigerian health facilities. We chose the year 2000 to begin the review as this allowed us to track trends through periods of more recent global prioritisation of reduction in maternal mortality.

There were no restrictions on the study design or language of publication for the inclusion of articles. However, studies were excluded if they were:

- Population-based surveys that reported on the primary outcomes of interest.
- Conducted in multiple countries from which the outcomes of interest for Nigeria could not be identified.
- Conducted during study periods that focused solely on periods before year 2000, irrespective of the year of publication.

### Search databases

We searched four electronic databases (African Journals Online (AJOL), Directory of Open Access Journals (DOAJ), EBSCOhost, and PubMed) for relevant literature. In addition, we searched the search engine Google Scholar for articles to be included for review. A preliminary search was conducted from October to December 2021 to test the pre-designed search strategy. Subsequently, a comprehensive search was conducted using the predetermined search terms between February 2022 to May 2022.

### Search strategy

Using a variant of the population-intervention-comparison-outcome (PICO) criteria—PIO [68], search terms were divided into three categories reflecting the key components of the research question:

- Population: "Nigeria"
- Intervention: "Caesarean section", "C-section", "Caesarean delivery", "Caesarean birth”, “Caesarean”, “CS”
- Outcome: "Prevalence", "Rate", "Trend", "Factors", "Outcomes", "Effects", "Impacts", "Complications", "Indications"

The search terms were combined using the Boolean operators: ‘AND' between concepts and 'OR' within concepts. Time-range filters were used to identify studies published from the year 2000 within the selected databases. The specific keywords used on each database are detailed in Additional file 1: Table S1. A review of reference lists of the retrieved articles was conducted to identify other relevant articles that may have been missed in the search process. When indicated, full versions of articles behind a paywall were purchased. The search was conducted independently by two authors (IO and OO), with search results compared for completeness.

### Identification and selection of studies

Two authors (IO and OO) independently screened the articles based on the pre-defined eligibility criteria, after which all authors agreed on the finality of the articles for the entirety of the review, particularly the meta-analysis. If the titles or abstracts were relevant, the full texts were subsequently reviewed to determine the eligibility of the articles for this review and the reasons for exclusions were documented. An automated reference manager, Mendeley Desktop V.1.19.4 V.2.74.0 (Elsevier, Amsterdam, The Netherlands), was used to store the full texts of the relevant articles to enhance accessibility for the review team.

### Quality assessment

The quality of each included study was ascertained using a modified Joanna Briggs Institute (JBI) Critical Appraisal Checklist. The JBI critical appraisal tool comprises different checklists, three of which are tailored to cross-sectional, case–control, and cohort studies [69]. These checklists were modified to include only those questions that evaluated the specific criteria being reviewed in this systematic review and meta-analysis, reducing the questions in each checklist to five. For every question, each article was scored one if it met the criterium but zero if it did not. The quality of the papers was deemed high if they met 100% of the set criteria, medium if they met 80 to < 100% of the set criteria, and low if they only met < 80% of the set criteria. Regardless of the assessed quality, all papers were included in this review to eliminate the risk of publication bias. The quality assessment was performed by OO and UG-A, with resolution of conflicts carried out by clarifying the given criteria against the different scores and discussing any observed differences. Where conflicts could not be resolved between these two assessors, they were settled by involving the senior author, AB-T.

### Data extraction and synthesis

Data extraction of relevant findings was conducted by two review authors (IO and OO) using pretested data extraction forms prepared on Microsoft Excel (Microsoft Corporation, Washington, USA). The data extracted included:

- The article description (title of publication, author(s), publication year, publication title, aim/objectives, reported study design, data sources, eligibility criteria, and period of study)
- Study setting (facility name, number of facilities, study location and geopolitical zone—North-East (NE), North Central (NC), North-West (NW), South-East (SE), South-South (SS), and South-West (SW))
- Health facility characteristics (ownership status and hierarchy of health care)
- Outcomes of interest (number of deliveries, number of CS, prevalence of CS, prevalence of emergency CS, prevalence of elective CS, indications, and complications)
- Participants’ specific data (booked patients, non-booked patients, mean age, age group, parity, gestational age, mean gestational age)

For multicentre studies, individual facility prevalence data were extracted. Indications and complications grouped as “others” by the authors of the included studies were not extracted if we could not identify them individually with their frequencies. Data were summarised using narrative synthesis. For prevalence data, the average CS rate over the study duration in each included study was extracted. CS rates in the included studies were computed using this formula: (Total number of caesarean deliveries/Total number of deliveries) × 100. All queries were resolved through consultations and team discussions.

### Meta-analysis

The reported prevalence rates for the overall, emergency, and elective CS were collated and coded into a dataset using Microsoft Excel (Microsoft Corporation, Washington, US). This dataset was then imported into RStudio (2022.02.3 Build 492 software Boston, Massachusetts, US), which was used for the meta-analysis. The distributions of the raw prevalence data were then tested for normality using the Shapiro–Wilk test. Thereafter, a logit transformation of the raw proportions, which is ideal when the observed proportions are either below 0.2 or more than 0.8 [70, 71] was performed. Confirmation of normality was achieved through the Shapiro–Wilk test.

Using the DerSimonian-Laird estimator, the random-effects model was applied to pool the prevalence of the overall intra-facility CS as well as that of the emergency and elective procedures from January 2000, owing to its ability to take the between-study (*τ*^2^) and within-study (v_*i*_) variances into consideration [71]. The study heterogeneity (*τ*^2^) was tested using the *χ*^2^ test with the *Q* statistic, while the proportion of the observed variability of the between-study variance was estimated using the *I*^2^ statistic. A forest plot was created to visually represent the study effects and their 95% confidence interval (CI).

Furthermore, using the mixed effects model, subgroup analyses of the intra-facility CS rates by region (north and south, based on the aggregation of all northern and southern geopolitical zones, respectively), study period (pre-WHO statement [2000–2014] and post-WHO statement [2015–2022]), class of facility service (secondary and tertiary), and type of facility (public and private/religious), were conducted to investigate the heterogeneity of the data. The selection of feasible sub-group analyses was based on the Cochrane guidelines for systematic reviews and meta-analyses, which recommended the inclusion of only a few groups specified in advance, which are being reported by at least ten included studies [72].

Thereafter, a forest plot was created to represent the study effects visually, their 95% CI and the pooled prevalence of CS in the two regions and two study periods, respectively. A scatter plot was also plotted to visualise the moderator effect of the different subgroups. Finally, publication bias was probed through a funnel plot, and any observations were confirmed by applying Egger’s regression test. IO conducted the meta-analysis.

### Narrative synthesis section

A two-step content analysis of the included studies [73, 74] was conducted to synthesise the evidence in the data on the indications and complications of CS in Nigeria. First, a manifest content analysis was conducted to identify and quantify the indications and complications of CS as reported in the included studies. This analysis was done by extracting frequencies and proportions of reported indications and complications for overall, elective, and emergency CS, as deemed relevant. Where authors presented only frequencies, these were converted to proportions. In instances in which authors used different terminology for the reported indications and complications, these were collapsed for simplicity. Indications and complications were presented in tables using Microsoft Excel’s colour grading tool to reflect relative proportions, with green for the least value, red for the highest value, and yellow for the middle value. The other cells are filled with gradient colours depending on their values. Next, a latent content analysis of these findings was carried out to segregate the data into subcategories (geopolitical zones, study periods, hierarchy of facility service and type of facility) and identify emerging patterns in the data. IO and AB-T conducted this analysis.

## Results

**Fig. 1:**
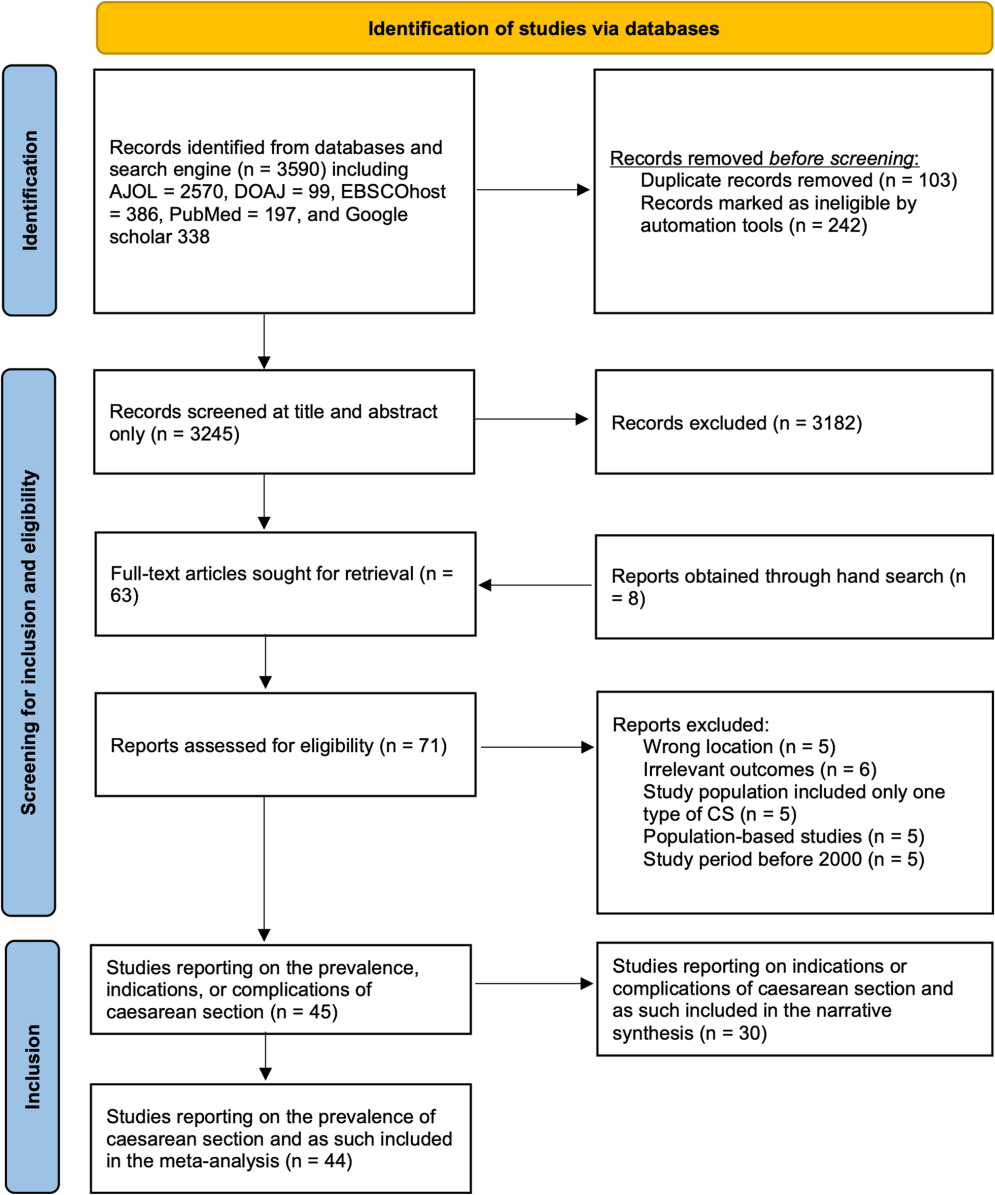
Flow diagram of literature search and results

From the 3590 records obtained from the initial search, the titles and abstracts of 3245 articles were screened for inclusion after duplicates were removed, and results were truncated by dates. The 63 articles that passed the screening and eight articles obtained by hand search were then assessed for eligibility. In all, 45 articles [21–65] were deemed eligible and as such included in the review (Fig. 1).

Forty-four of these were included in the meta-analysis, having all reported on CS prevalence [21–63, 65]. However, only 31 articles [21–24, 27, 28, 30–32, 35, 37, 38, 41, 42, 44–46, 48–50, 55–61, 63, 64] reported the indications or complications of CS and, as such, were included in the narrative synthesis. These are presented in the PRISMA flow diagram (Fig. 1).

### Study characteristics

Almost all included studies (41 [91.1%]) applied cross-sectional study design [21, 22, 24–28, 30–57, 65]. Of all the included studies, 27 (60.0%) were conducted in health facilities situated in the southern states [21, 23–25, 28, 29, 32, 34, 36, 38–40, 44–47, 49–53, 56, 57, 59, 60, 63, 65] while 17 (37.7%) were in health facilities situated in the northern states [22, 26, 27, 30, 31, 33, 35, 37, 41–43, 48, 55, 58, 61, 64]. Also, 82.2% of the included studies were conducted in public [21–38, 40, 42, 44–55, 57, 58, 60–62, 64, 65] and 80.0% were conducted in tertiary [21–40, 42, 44–47, 49–55, 57, 58, 60–62, 65] facilities. Also, 32 (71.1%) studies [21–44, 58–64] were conducted between 2000 and 2014 (pre-WHO CS statement), while eight (17.8%) studies [45–52] were conducted after the WHO statement was published (i.e., from 2015). The remaining five (11.1%) studies [53–57] had their study periods overlapping both timeframes (Table 1). A comprehensive presentation of the characteristics of included studies is available in Additional file 2.

**Table 1:** Summary of characteristics of the included studies

Characteristics	Number (N = 45)	Percentage (%)
*Study design*		
Cross-sectional	41	91.1
Case–control	1	2.2
Cohort	3	6.7
*Geopolitical zone*		
North-east (NE)	2	4.4
North-west (NW)	9	20.0
North-central (NC)	6	13.3
South-east (SE)	8	17.8
South-south (SS)	9	20.0
South-west (SW)	10	20.2
Multiple zones	1	2.2
*Hierarchy of facility service*		
Tertiary	36	80.0
Secondary	7	15.6
Mixed	1	2.2
Not defined	1	2.2
*Facility type*		
Public	37	82.2
Private/religious	6	13.3
Mixed	2	4.4
*Study period*		
2000–2014 (pre-WHO statement)	32	71.1
2015–2022 (post-WHO statement)	8	17.8
Overlaps both periods	5	11.1

### Quality of the included studies

Based on the findings from the quality assessment, 29 (64.4%) articles [23–27, 33–35, 37–39, 42, 44–46, 48–60, 63] were of high quality. Of the remaining, ten (22.2%) studies were of medium quality [21, 22, 29, 30, 36, 41, 43, 47, 65] and six (13.3%) were of low quality [31, 32, 40, 61, 62, 64], largely due to a failure to adequately describe study subjects and settings. Other reasons were poor use of statistical analytical methods and a lack of standard criteria for measurements used (Table 2).

**Table 2:**
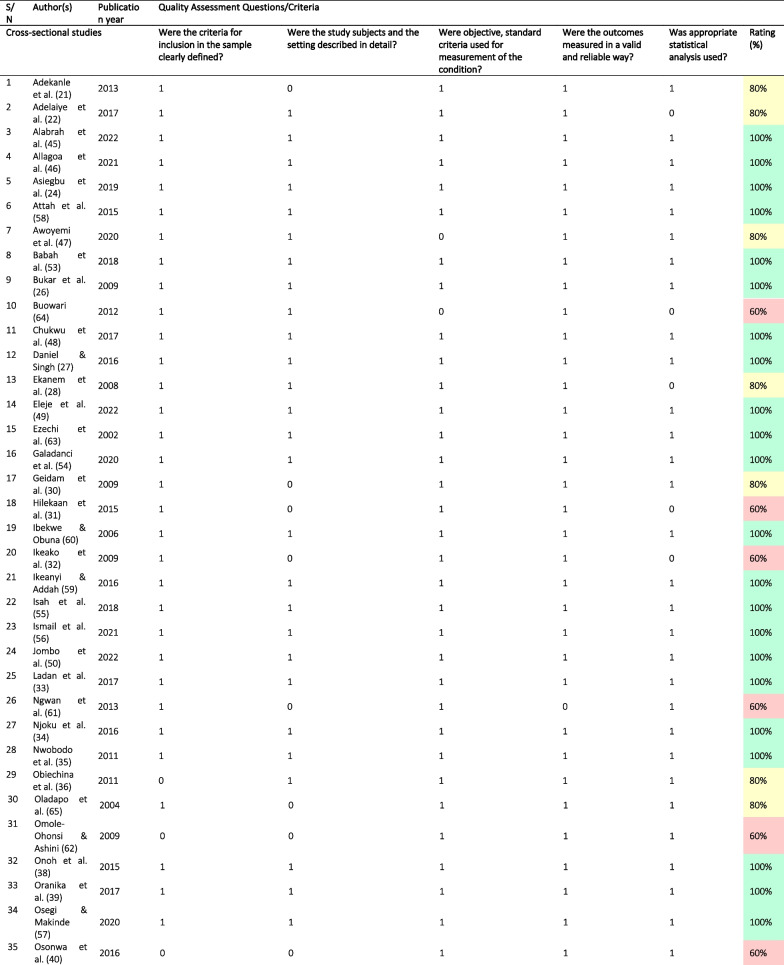

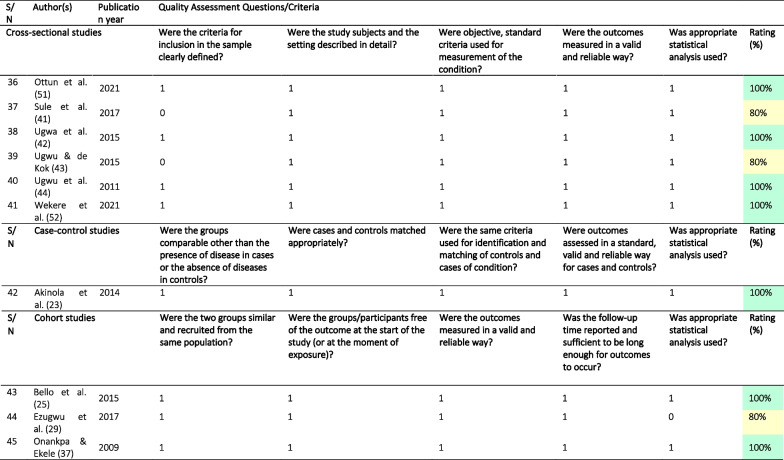
Quality assessment of the included studies

Rating was high (green) if they met 100% of the set criteria, medium (yellow) if they met 80 to < 100% of the set criteria, and low (red) if they only met < 80% of the set criteria

### Pooled prevalence of intra-facility caesarean section in Nigeria

The intra-facility prevalence of CS in Nigeria was estimated from a total of 459,612 deliveries in 70 facilities identified from 44 studies [21–63, 65] published from 2000 to 2022, with their study periods between 2000 and 2021. The raw proportions (W = 0.94 *p*-value < 0.01) deviated less further from a normal distribution after logit transformation (W = 0.96, *p*-value = 0.03). The prevalence of elective and emergency CS in facilities was reported from 33 studies [21, 22, 24–30, 32–42, 44, 45, 48, 50, 52, 55–60, 62, 65].

#### Overall caesarean section

**Fig. 2:**
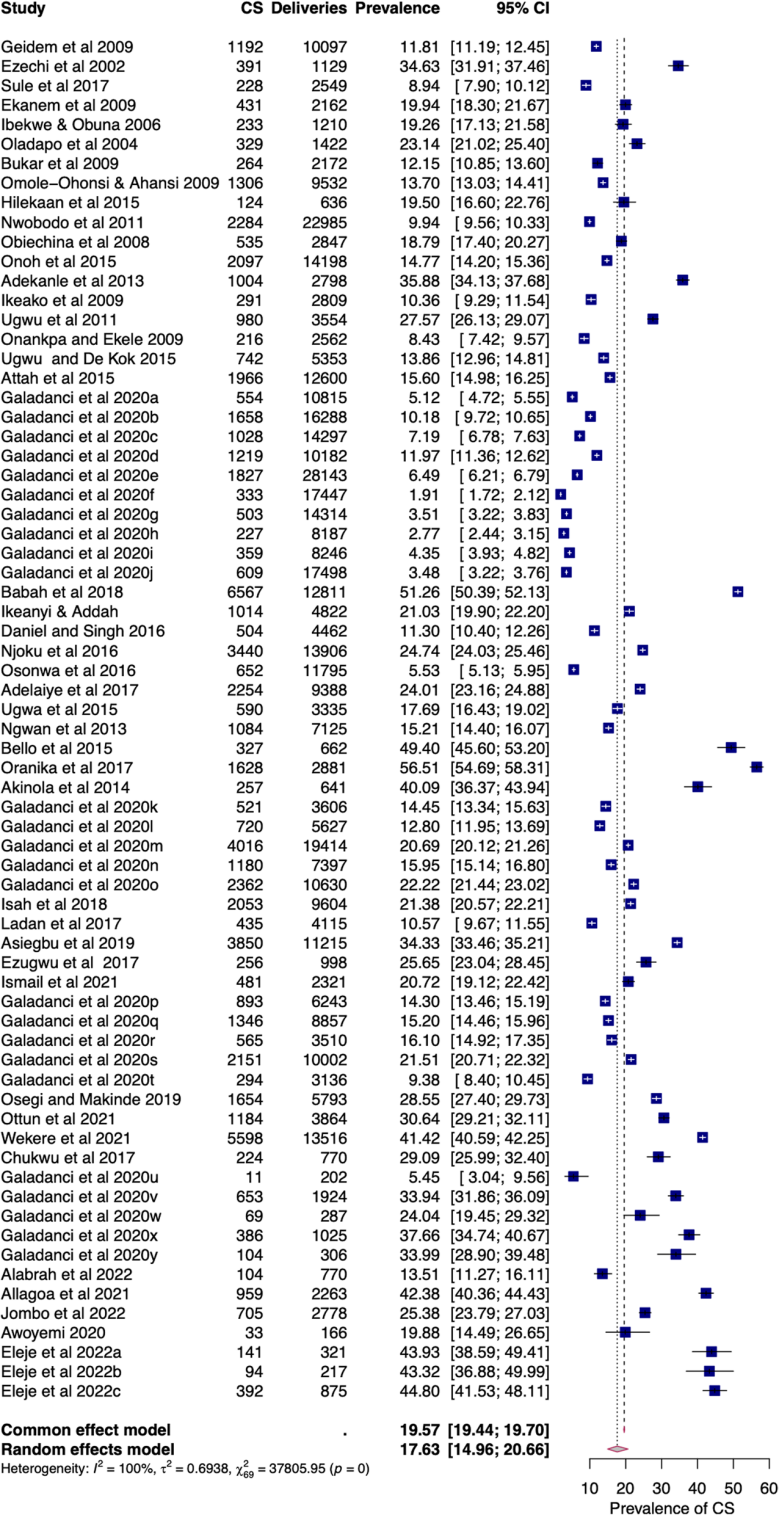
Forest plot of pooled prevalence of overall intra-facility caesarean section in Nigeria (2000–2022)

The overall prevalence of CS in health facilities across Nigeria was 17.63% (95%CI = 14.96–20.66), with a substantial level of heterogeneity (I^2^ = 99.82%) due to true differences between studies rather than chance (Fig. 2). This heterogeneity was first identified by Cochran's Q test (Q = 37,805.95, df = 69, *p* < 0.01), and the estimated heterogeneity (τ^2^) was 0.69 (95%CI = 0.60–1.18). However, the leave-out-one study analysis and Cook’s distance failed to identify any influential study (Additional file 1: Fig. S1).

#### Elective caesarean section

**Fig. 3:**
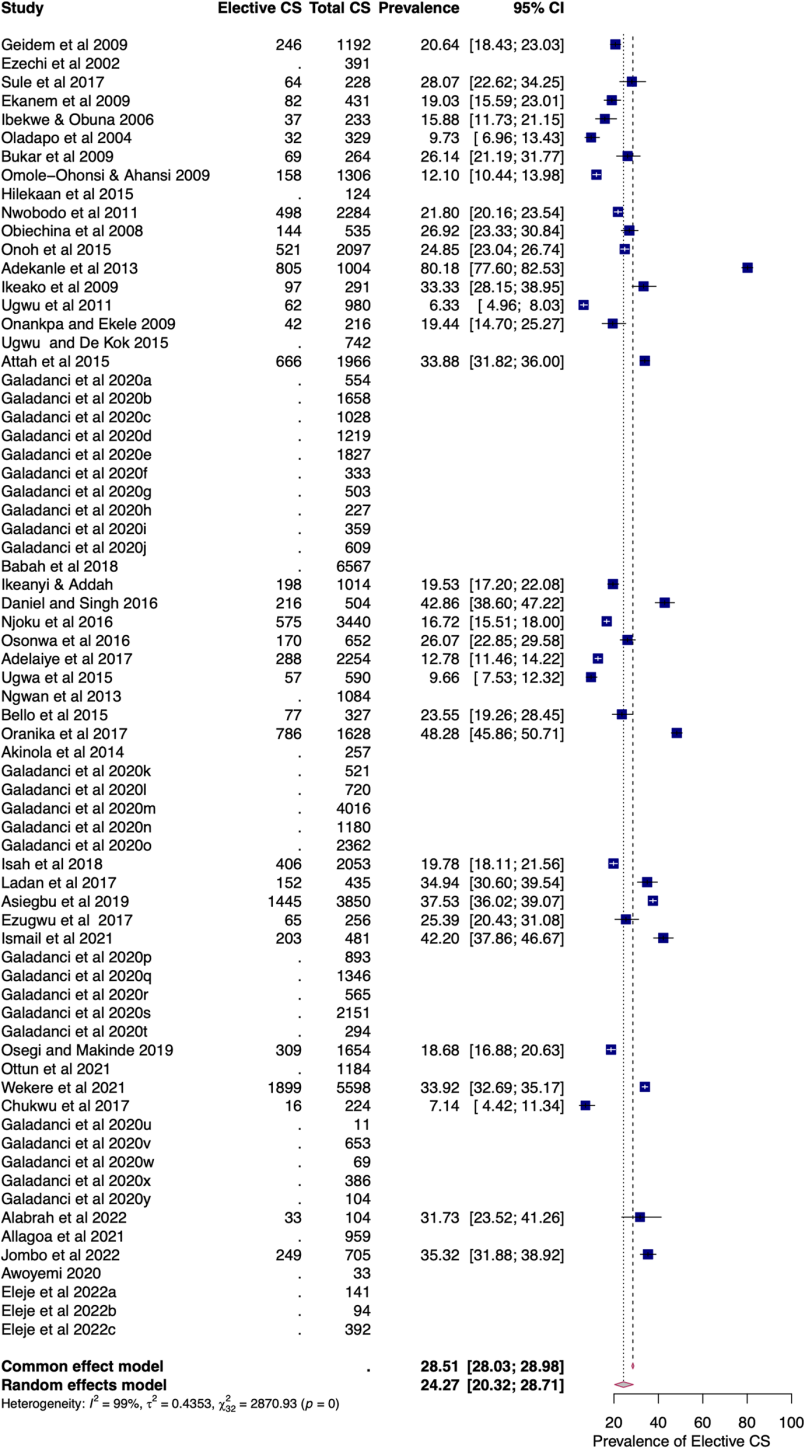
Forest plot of pooled prevalence of intra-facility elective caesarean section in Nigeria (2000–2022)

The pooled prevalence of elective CS amongst elective CS conducted in Nigerian health facilities was 24.27% (95%CI = 20.32–28.71). There was a substantial level of heterogeneity (I^2^ = 98.89%) due to true differences between studies rather than chance (Fig. 3). This heterogeneity was first identified by Cochran's Q test (Q = 2870.93, df = 32, *p* < 0.01), and the estimated heterogeneity (τ^2^) was 0.44 (95%CI = 0.38–1.07). The pooled prevalence of elective CS was 20.64% (95%CI = 16.07 to 26.11) and 26.87% (95%CI = 21.37 to 33.20) in health facilities in the north and south of Nigeria, respectively (Additional file 1: Fig. S2). In the north, the range of elective CS was 10–43% in tertiary facilities and 7–28% in secondary facilities. In the south, the range of elective CS was 6–80% in tertiary facilities and 20–42% in secondary facilities.

#### Emergency caesarean section

**Fig. 4:**
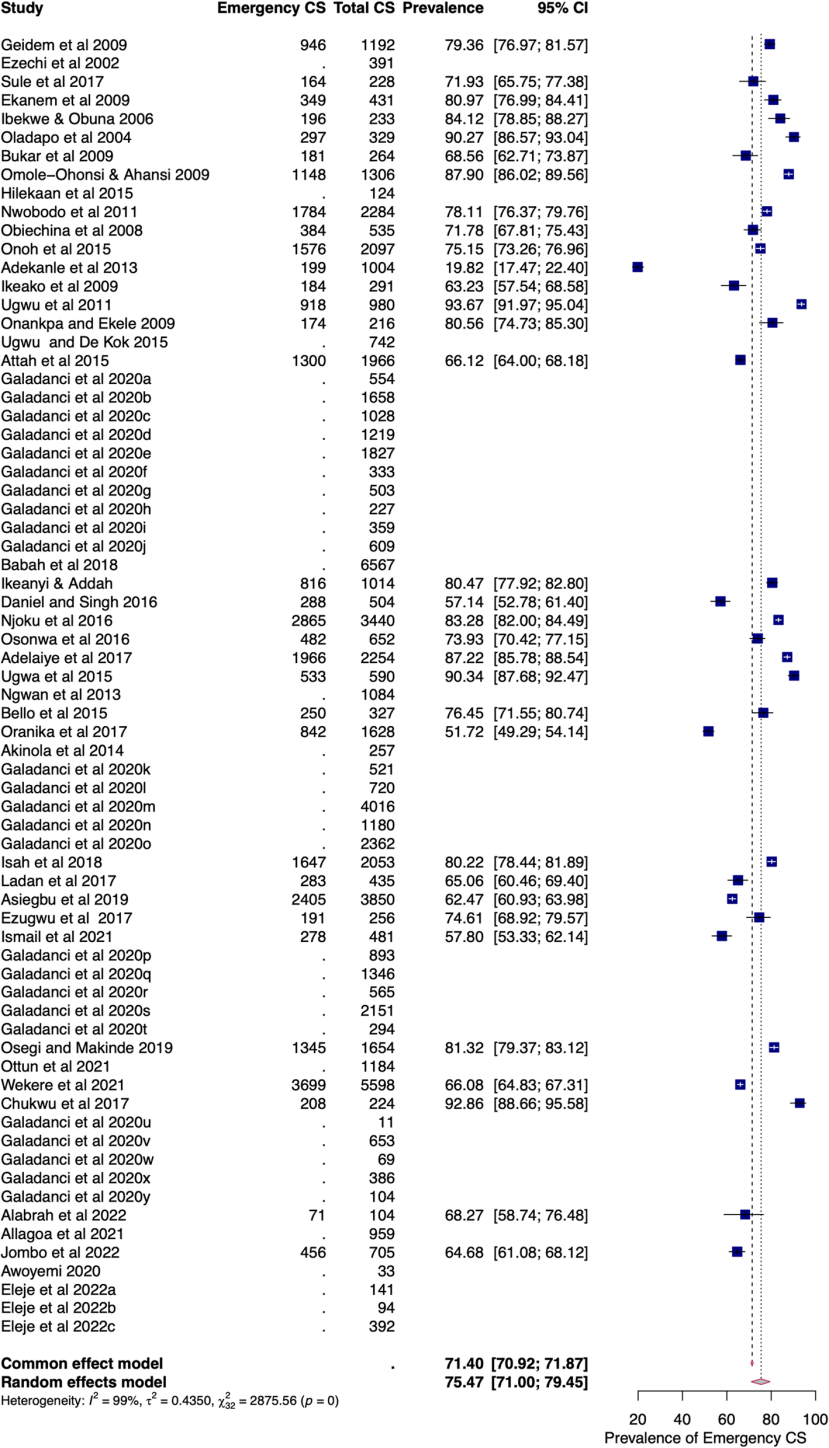
Forest plot of pooled prevalence of intra-facility emergency caesarean section in Nigeria (2000–2022)

The pooled prevalence of emergency CS amongst overall CS in Nigeria was 75.93% (95%CI = 71.40–79.95). There was a substantial level of heterogeneity (I^2^ = 98.93%) due to true differences between studies rather than chance (Fig. 4). This heterogeneity was first identified by Cochran's Q test (Q = 2989.63, df = 32, *p* < 0.01), and the estimated heterogeneity (τ^2^) was 0.46 (95%CI = 0.42–1.15). The pooled prevalence of emergency CS was 79.03% (95%CI = 73.46 to 83.68) and 72.91% (95%CI = 66.56 to 78.45), in the north and south of Nigeria, respectively (Additional file 1: Fig. S2). In the north, the range of emergency CS was 57–90% in tertiary and 72–93% in secondary facilities. In the south, the range of emergency CS was 20–94% in tertiary and 57–81% in secondary facilities.

### Pooled prevalence by subgroups

**Fig. 5:**
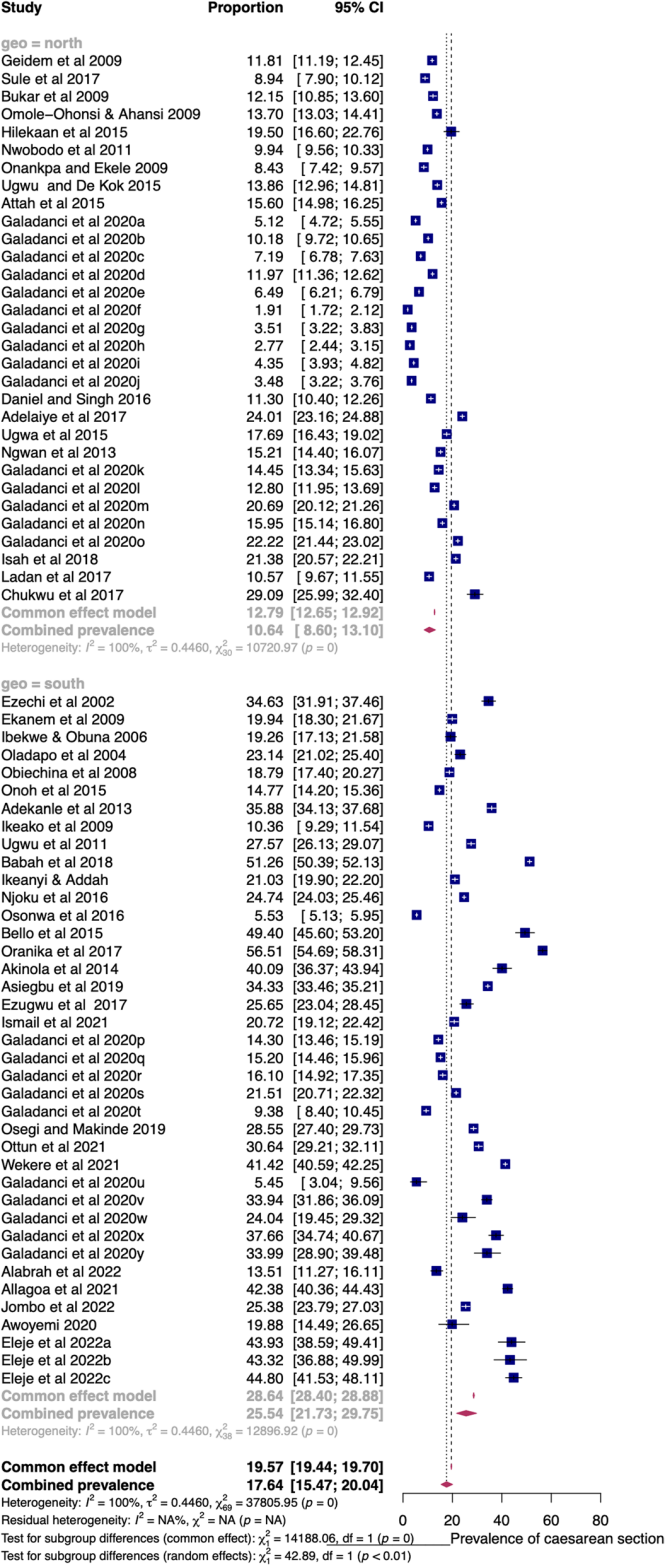
Forest plot of pooled prevalence of CS in health facilities in northern and southern Nigeria (2000–2022)

In health facilities in the northern and southern states of Nigeria, the pooled prevalence of CS was 10.64% (95%CI = 8.60 to 13.10) and 25.54% (95%CI = 21.73 to 29.75), respectively (Fig. 5). The difference between the summary estimates of the prevalence in northern and southern facilities was statistically significant (QM(1) = 42.89, *p* < 0.01). The significant heterogeneity between the facilities (QE(68) = 23,617.89, *p* < 0.01) was partly moderated by their geopolitical zone (R^2^ = 35.72%). The significant regression coefficient (1.06; Z(68) = 6.55; *p* < 0.01) confirmed geopolitical zone as a significant moderator of the prevalence of CS in Nigeria.

The pooled prevalence before the WHO’s recommendation was 18.90% (14.94–23.61), but this increased to 29.63% (21.97–38.65) afterwards (Additional file 1: Fig. S3). The study period significantly moderated (QM(1) = 8.99, *p* < 0.01) the prevalence of CS, and this was confirmed by the significant regression coefficient (0.60; Z(47) = 3.00, *p* < 0.01) (Additional file 1: Fig. S4). However, the hierarchy or level of facility service (tertiary or secondary) was not a significant moderator (QM(1) = 1.17, *p* = 0.28) of the prevalence of CS, and this was supported by the insignificant regression coefficient (− 0.24; Z(52) =  − 1.08, *p* = 0.28) (Additional file 1: Fig. S5). Also, the type of facility (public or private) was not a significant moderator (QM(1) = 0.33, *p* = 0.57) of the prevalence of CS, with an insignificant regression coefficient (0.16; Z(47) = 0.57, *p* = 0.57) (Additional file 1: Fig. S6).

### Publication bias

**Fig. 6:**
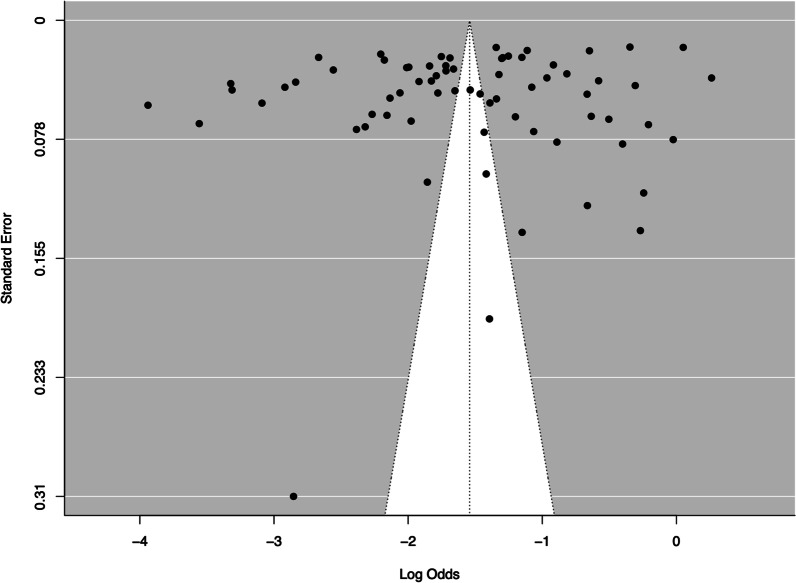
Funnel plot to investigate publication bias

The symmetrical distribution of the funnel plot was confirmed using the unweighted Egger's regression test for funnel plot asymmetry. This showed that there was no statistically significant publication bias present (z = – 0.07, *p* = 0.94) (Fig. 6).

### Indications for caesarean section in Nigeria

From the 28 included studies that reported indications [22–24, 30–32, 37, 38, 41, 42, 44–46, 48–50, 55–64], previous scar/CS (3.5–33.5%) and hypertensive disorders in pregnancy (5.5–30.0%) were the most common indications for CS, with 22 (84.6%) of these studies reporting both. Other common indications were cephalopelvic disproportion (CPD), foetal distress, obstructed labour antepartum haemorrhage and multiple pregnancies, with at least 15 (57.7%) studies reporting their occurrence. Disaggregated by regions, previous scar/CS (6.1–27.8%) was the most prevalent indication among the southern facilities, while CPD (2.0–39.9%) was the most prevalent indication in the northern facilities. Within the facilities in the northern geopolitical zones, isolated cases of a high prevalence of obstructed labour (48.4%) and foetal distress (40.4%) occurred in the north-west and north-east zones, respectively [30, 64]. In the south, CPD was distinctly prevalent in three studies in the south-south (31.9%, 36.0%) and south-west (32.8%) [46, 59, 63] (Table 3). The comprehensive compilation of the indications retrieved from the included studies is available in Additional file 2.

**Table 3:**
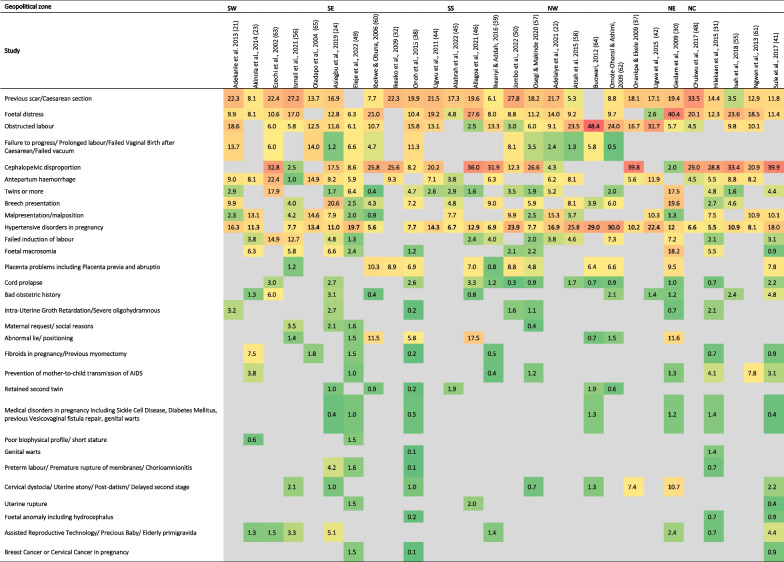
Indications for CS in health facilities across the geopolitical zones in Nigeria

The table includes data from only articles that reported overall CS indications, not split by elective or emergency CS. Indications and complications were presented in tables using Microsoft Excel’s colour grading tool to reflect relative proportions, with green for the least value, red for the highest value, and yellow for the middle value. The other cells are filled with gradient colours depending on their values.

### Complications of caesarean section in Nigeria

From 20 of the included studies [24, 27, 28, 30, 32, 35, 37, 38, 44–46, 48–50, 55, 58, 59, 62–64], anaemia, wound sepsis/dehiscence, maternal death and perinatal death were the most reported complications of CS. Anaemia (6.4–57.1%) was the commonest complication following CS across northern and southern facilities, being more prevalent in the emergency procedures (2038–60.2%) compared to the elective cases (13.7–36.9%). Disaggregated by zones, postpartum haemorrhage was highest (20.5–59.7%) in the north-western facilities, while anaemia (6.4–57.1%) was the most prevalent in the south-southern facilities. Maternal death remained low across all studies (0.5–3.6%) following CS, except in one south-western private facility where the rate was 6.1% [63]. Perinatal death was highly prevalent (11.1–18.5%) following CS as well as after the elective (16.7%) and emergency cases (19.0%) in north-western facilities. All but four [48, 59, 62–64] of these facilities reporting on the complications were tertiary ones (Table 4). A comprehensive compilation of the complications of CS as retrieved from the included studies is available in Additional file 2.

**Table 4:**
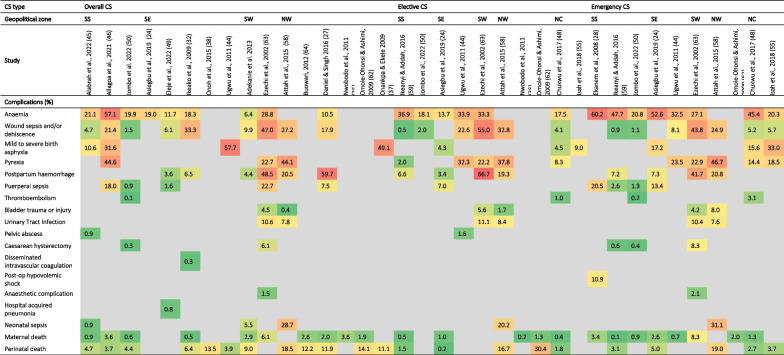
Complications following caesarean section in health facilities in different geopolitical zones of Nigeria

Indications and complications were presented in tables using Microsoft Excel’s colour grading tool to reflect relative proportions, with green for the least value, red for the highest value, and yellow for the middle value. The other cells are filled with gradient colours depending on their values. Conditions that would otherwise not be established complications of CS such as birth asphyxia and low-birth weight as complications were excluded, even if reported by authors of the included studies

## Discussion

We set out to pool the prevalence of CS in facilities across Nigeria, spanning the years 2000 to 2022. We retrieved 45 papers in total, with most of them (29 (64.4%)) being of high quality. We obtained an overall prevalence of CS of about 17.6% and identified a higher prevalence of emergency CS amongst overall CS (75.9%) compared to elective CS (24.3%). We also identified a significantly higher prevalence of CS in the southern facilities (25.5%) compared to the northern ones (10.6%). Furthermore, we observed an increase in the prevalence of intra-facility CS in the country following the WHO statement on CS rates (from 18.9 before to 29.6% after). However, none of the reporting studies adopted the Robson classification of CS to determine intra-facility CS rates. In addition, neither the hierarchy of care (tertiary or secondary) nor the type of facility (public or private) significantly influenced the prevalence of CS. The most common indications for a CS were previous scar/CS, CPD, foetal distress and hypertensive disorders in pregnancy. On the other hand, anaemia and wound dehiscence were the most common complications reported in the included studies.

At 17.6%, we found the overall pooled prevalence of CS in health facilities across Nigeria to be high when compared to the institutional CS rate reported in similar West African countries like Sierra Leonne (2.9%) and Cameroon (9.9%) [75, 76]. In the 2015 Lancet series on CS, the intra-facility CS rate was estimated as 7.2 per 100 live births across West and Central Africa and 11.1 per 100 live births across Eastern and Southern Africa [13]. Within the country, we found regional differences in the prevalence of CS in Nigerian health facilities. In the south, the pooled intra-facility prevalence of CS (25.5%) was more than twice the prevalence recorded in the north (10.6%). Although more studies were conducted in the south (60.0%), this vast difference in intra-facility CS prevalence raises questions on varying accessibility and utilisation in the north and south regions of the country. When disaggregated by type of CS within the regions, there was a non-significantly higher pooled prevalence of elective CS in health facilities of the south (26.87% (95%CI = 21.37 to 33.20)) compared to the north (20.64% (95%CI = 16.07 to 26.11)). On the other hand, there was a non-significantly higher pooled prevalence of emergency CS in health facilities in the north (79.03% (95%CI = 73.46 to 83.68)) compared to those in the south (72.91% (95%CI = 66.56 to 78.45)).

Based on facility-type, tertiary facilities were the most dominant study settings (80.0%) among the included studies in our review. However, we found the reported prevalence rates in tertiary facilities (5.5–56.5%) to be broadly comparable with the prevalence rates in secondary facilities (5.4–37.7%), with only nine [23, 25, 39, 46, 49, 52, 53] of the 70 tertiary facilities included in the review recording intra-facility CS rates greater than the range observed in secondary facilities. A similar pattern of comparable intra-facility CS prevalence in secondary (10.1% (95% CI: 5.1, 16.6%)) and tertiary hospitals (15.4% (95% CI: 12.5, 18.6%)) was reported in a Cameroonian systematic review [75]. This reality conflicts with the general expectation that tertiary facilities should conduct more CS owing to the high referral load of complicated obstetric emergencies from private hospitals and public secondary facilities [77]. In our review, when the data was disaggregated by hierarchy of facility, there was no difference in the rates of elective CS in tertiary facilities (9.6–42.9%) v. secondary facilities (19.5–48.3%) and rates of emergency CS in tertiary facilities (57.1–90.4%) v. secondary (51.7–80.5%).

Similarly, our analysis showed that the type of health facility (public or private) did not significantly influence the intra-facility CS rate. However, caution might be needed with this finding, as we only found six studies conducted in nine private facilities, despite the higher preference for private healthcare reported in Nigeria [78–82]. In our review, we observed a higher prevalence of CS (20.7–56.5%) in private facilities in the southern region [39, 54, 56, 59, 63]. This finding mirrors previous reports that indicate a higher prevalence of CS in private hospitals in Nigeria and other African countries [83]. However, much lower prevalence rates (8.9–13.9%) were recorded in private facilities in the northern region [41, 43]. Economic constraints from direct and indirect costs of the surgery in private settings have been reported to be major contributors to decreased patronage as private facilities charge more for CS than public facilities in many low- and middle-income countries and may explain this observation in the north of Nigeria [84]. This is a very important consideration for many women in this region who live below the poverty line, as even within the public sector, it is not uncommon to pay over US$400 for a CS [85]. In addition, the poor distribution of health workers in the north may limit the capacity of skilled health personnel in the private sector of northern Nigeria to render these services [86].

At 29.6%, the prevalence of CS in facilities in Nigeria, following the release of the WHO’s statement in 2015, showed a 1.6-fold increase. This was despite our retrieval of fewer studies (17.8%) post-2015 than those published from 2000 to 2014 (71.1%). However, we identified overlapping confidence intervals of the prevalence rates of CS in the years before (18.9% [95%CI = 15.0–23.6]) and after (29.6% [95%CI = 22.0–38.7]) the release of the WHO statement. Hence, although the study period was a significant moderator of the prevalence of CS in our review, we cannot conclude that the increase in CS post-2015 is statistically significant. We also found that post-2015, more research emerged from southern facilities. At the same time, there was a noticeable lack of research in the north as only one study [48] was situated in this region during this period. It is entirely plausible that this dominance of studies from the southern facilities is a major contributor to the marked increase in the prevalence of CS in facilities post-2015. However, further research is needed to enable a comprehensive characterisation of intra-facility CS in the northern region.

Another critical time-related variation in intra-facility CS rates worth flagging relates to the pandemic of Coronavirus disease 2019 (COVID-19). Only one article in our study reported this, finding that intra-facility CS rate during the COVID-19 pandemic in three tertiary hospitals in the South-East was significantly lower than the period prior to the pandemic (40.0% vs. 46.8%; *p* = 0.027) [49]. This is similar to the reduction seen especially during the first wave of the COVID-19 in another teaching hospital in the South-West of Nigeria [87].

We found the widely reported indications for CS to be previous CS/scar (3.5–33.5%), hypertensive disorders in pregnancy (5.5–29.0%), foetal distress (2.6–40.0%), CPD (2.0–39.9%) and obstructed labour (2.5–44.4%), across all studies and geopolitical zones. These trends are similar to those obtained from many other African countries where previous CS and obstructed labour are common indications for CS [88]. These indications, excluding a previous CS/scar, fall within the scope of obstetric emergencies and may explain the higher prevalence of emergency CS in the country. We found that emergency CS were three times more prevalent (75.9%) than the electives (24.3%). Particularly, more women presented with CPD (20.9–39.9%) in the north-central facilities. Also, there was an isolated high occurrence (40.4%) of foetal distress in the north-east [30]—a zone particularly burdened with security issues [89]. Such insecurities may prevent women from accessing safe obstetric care and lead to late presentations. Similar issues have been observed in the highly conflicted Tigray region in Ethiopia when compared to other parts of the country [90]. Finally, other factors have been reported to inhibit access to CS in health facilities in the north, including inhibitory cultural practices, low socio-economic statuses, poor or no formal education, poor attendance at antenatal clinics, high-risk home deliveries, and permission needed from a spouse to undergo surgery, to mention a few [16, 91, 92]. These factors might suggest that women are unlikely to access medicalised birth in health facilities early enough and only present when situations have deteriorated, which warrants an emergency CS.

Regarding complications, anaemia was the most reported complication across facilities (10.5–57.1%) and even more so in emergency CS (20.8–60.2%). The high prevalence of postpartum anaemia in African countries is reportedly due to poor haemoglobin levels prior to delivery (resulting from poor nutritional intake and non-adherence to routine haematinics) and postpartum haemorrhage [93]. Although less commonly reported, postpartum haemorrhage following CS was the most frequent complication (59.7%) in one report from a tertiary facility in northern Nigeria [27]. Per a 2019 review, postpartum haemorrhage caused a third of all deaths following CS [94, 95]. Pyrexia was also a common complication, irrespective of the type of CS (8–45%) [45, 58, 63]. A probable reason for this could be the choice of anaesthesia, as spinal and epidural anaesthesia have been associated with postpartum pyrexia [96]. For maternal death associated with overall CS, this ranged from 0.5 to 3.6% across both north and south regions, with an outlier of 6.1% in a south-western private facility [63]. The non-emergence of a clear north–south pattern as it relates to the complication of maternal death with CS might suggest that the higher prevalence of maternal mortality in the north compared to the south of Nigeria may have more to do with maternal deaths occurring more because of issues within the community, as opposed to the facility [97]. On the contrary, there was a seeming pattern of higher proportions of perinatal deaths in health facilities in the northern region compared to the south.

Our review is novel because we have successfully conducted the pooled intra-facility prevalence of CS across Nigeria and identified the key indications and complications of the procedure among women undergoing CS in the country. In terms of policy, the constellation of findings pointing to generally higher emergency CS rates, higher rates of foetal distress, and higher perinatal deaths in the north are suggestive of delays in the presentation of pregnant women to health facilities. There is a need to promote access to and use of CS in the northern facilities by addressing factors that increase the risk of an obstetric emergency. In southern facilities, higher rates of previous scar/CS as an indication for a CS, compared to the north, suggests some overuse of the procedure in the south. More consideration needs to be given to alternative delivery options, such as assisted vaginal delivery whose use remains significantly low in many low- and middle-income countries [98]. This will come at an additional cost for service provision; however, emphasis must remain on ensuring value for money [99, 100]. Our finding of comparable intra-facility CS rates between tertiary and secondary facilities suggests that capacity at both facility levels may not be significantly different. Referral decisions may need to consider institutional capacity irrespective of the secondary or tertiary status of receiving facilities rather than ‘climbing up the hierarchy ladder’ for all complicated cases [101]. These would help to reduce the workload that skilled health personnel tackle in tertiary institutions. It may also encourage the equipping of more secondary hospitals to offer these services.

Regarding research, the practice of comparing intra-facility rates with the WHO population-based recommendations of CS [15], as done in some of the included studies [22, 23, 27], needs to be discouraged. This change in practice will significantly improve the capacity for these studies to inform recommendations for practice. In addition, none of the studies we retrieved for this review used the Robson classification, as recommended by the WHO. This is being adopted by studies conducted in other African countries like Egypt, Tanzania, and Ghana [77, 102, 103] and can certainly be done in Nigeria, especially as a number of studies included in our review already report some of the parameters needed for the classification system (as seen in Additional file 2). They just do not report CS rates within groups defined by these parameters. Also, there is a need for more transparency with conducting CS provision research, especially when being conducted in private hospitals. Simply describing the setting as a “private” facility or not indicating the state where the facility is located as done in some included studies [41, 43, 54] hinders capacity for comparison in future assessments and tracking progress. Finally, there is a need for more research, particularly in the north-eastern parts of Nigeria as well as in secondary and private health facilities.

In terms of strengths, our review is the first to pool prevalence of intra-facility CS in Nigeria, aggregating 45 studies, 64.4% of which were rated high quality, thereby increasing the reliability of our findings. Another key strength is our presentation of rates disaggregated CS type, region, as well as type and hierarchy of facility. However, despite our attempt to ensure a robust analysis of trends in the prevalence, indications and complications of CS in Nigerian hospitals, this review has limitations. First, despite our best effort, we were only able to retrieve a few studies conducted in secondary and private facilities, and the north-east zone was poorly represented. Second, there was a lack of uniform terminology for indications and complications across studies, and in other instances, the frequencies of multiple indications and complications were aggregated or ambiguously classified as ‘others’. Also, because we reported indications and complications as reported in the individual studies, we were unable to specify if co-indications or co-complications were prevalent. However, our use of a multi-disciplinary team, that included clinicians, allowed us to critically review and reclassify original classifications made by authors of the included studies, thereby minimising errors that could arise as much as possible.

## Conclusion

Our review estimated the pooled prevalence of intra-facility CS in Nigeria to be 17.6%, which is high compared to estimates from many countries in the African region. Within the country, there are variations in CS prevalence, indications, and complications suggestive of geographical inequities, CS overuse, which is a waste of limited human, capital, and financial resources, and underuse which leads to poorer health outcomes and may contribute to increased maternal deaths. This lack of optimal use of CS could set Nigeria further back in its effort to reduce maternal and perinatal mortality in line with the relevant targets of Sustainable Development Goal 3. Implementation of comprehensive solutions for optimisation of CS provision and utilisation tailor-made for health facilities in the north and south regions of Nigeria will help drive the much-needed change.

## Supplementary Information

**Additional file 1: Table S1.** Keywords for databases. **Figure S1.** Investigating the presence of influential studies. **Figure S2.** Forest plot of the prevalence of caesarean section by region and type of caesarean section. **Figure S3.** Forest plot of the prevalence of caesarean section prior to and after the WHO statement. **Figure S4.** Scatter plot to show the moderating effect of study period. **Figure S5.** Scatter plot to show the moderating effect of the class of facility service. **Figure S6.** Scatter plot investigating the moderator effect of the type of facility.

**Additional file 2.** Data extraction sheet.

## Data Availability

The datasets used for this review are available in the additional files.

## Abbreviations

CI: Confidence interval
CPD: Cephalopelvic disproportion
CS: Caesarean section
EmOC: Emergency Obstetric Care
JBI: Joanna Briggs Institute
NDHS: National Demographic and Health Surveys
PRISMA: Preferred Reporting Items for Systematic Reviews and Meta-Analyses
WHO: World Health Organization
