# Supplementary material for: Prevalence, indications, and complications of caesarean section in health facilities across Nigeria: a systematic review and meta-analysis
Source: Reprod Health. 2023 Jun 2;20:81. doi: 10.1186/s12978-023-01598-9 (PMC10237076; doi:10.1186/s12978-023-01598-9)

Additional file 1

Table S1. Keywords for databases

The final keyword combinations on the different databases are as follows:

1. PubMed: 197 HITS<br>Keywords were combined using “I” AND “II” AND “III” AND “IV”.<br>Where:<br>I: “Cesarean section OR Cesarean delivery OR Caesarean section OR Caesarean delivery OR C-section OR CS”<br>II: “Prevalence” OR “Rate” OR “Trend”<br>III: “Outcomes” OR “Effects” OR “Impacts” OR “Consequences” OR “Complications”<br>IV: “Nigeria"
2. African Journals Online (AJOL)<br>“Prevalence”, “Complication”, “Indication”, “C-section”, “Nigeria” 2110 HITS<br>“Caesarean Section” Indications Prevalence Outcome “Nigeria” 460 HITS
3. Directory of Open Access Journals (DOAJ)<br>‘Indications OR Prevalence OR Complications’ “Caesarean Section" "Nigeria" 99 HITS
4. Google Scholar<br>“Prevalence of caesarean” AND “Outcome” AND “Nigeria” 192 HITS<br>“Prevalence of caesarean” AND “indications” AND “Nigeria” 146 HITS
5. EBSCOhost<br>“Prevalence of caesarean” AND “Outcome” AND “Nigeria” 120 HITS<br>“Prevalence”, “Complication”, “Indication”, “C-section”, “Nigeria” 155 HITS<br>“Prevalence of caesarean” AND “complication” AND “Nigeria” 45 HITS<br>“Prevalence of caesarean” AND “indications” AND “Nigeria” 66 HITS

Influential analysis

The leave-one-study-out analysis was used to determine if a single study could significantly influence the pooled prevalence of caesarean section (CS) in this review. The absence of a red point in Cooks distance measure indicates the absence of any influential studies.

Figure S1. Investigating the presence of influential studies

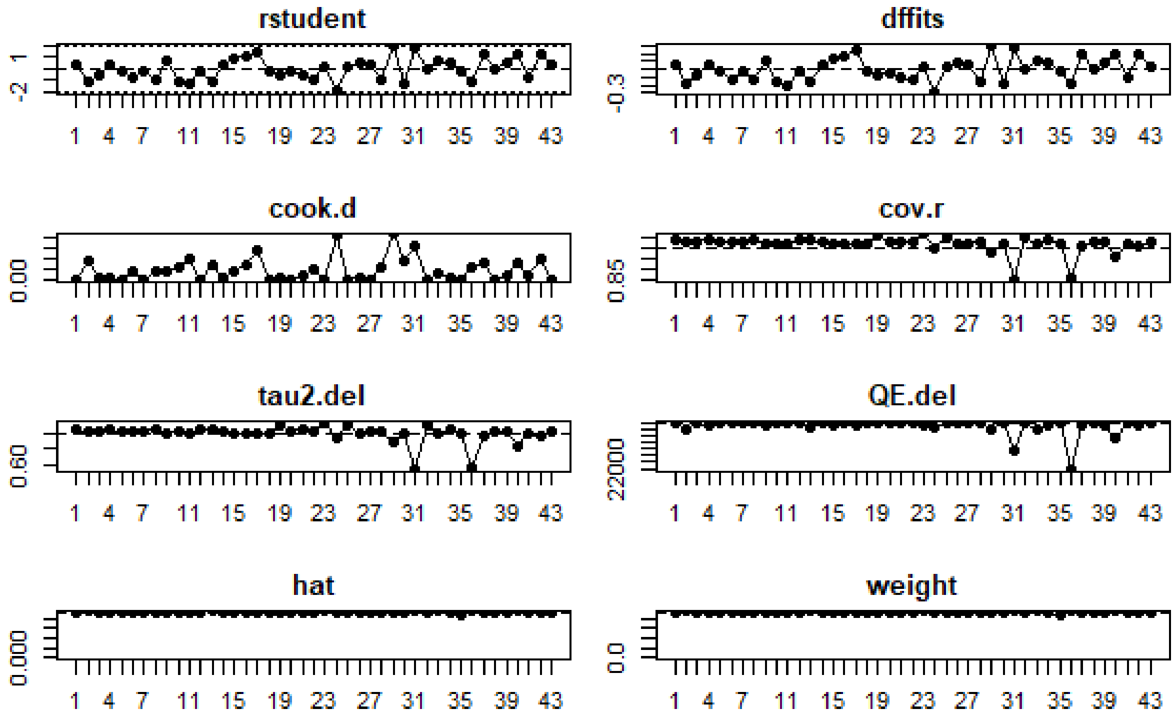

Region (North and South) and type of caesarean section (Elective and Emergency)

The pooled prevalence of elective CS was 20.64% (95%CI=16.07 to 26.11) and 26.87% (95%CI=21.37 to 33.20) in health facilities in the north and south of Nigeria, respectively. On the other hand, the pooled prevalence of emergency CS was 79.03% (95%CI= 73.46 to 83.68) and 72.91% (95%CI= 66.56 to 78.45), in the north and south of Nigeria, respectively.

Figure S2: Forest plot of the prevalence of caesarean section by region and type of caesarean section

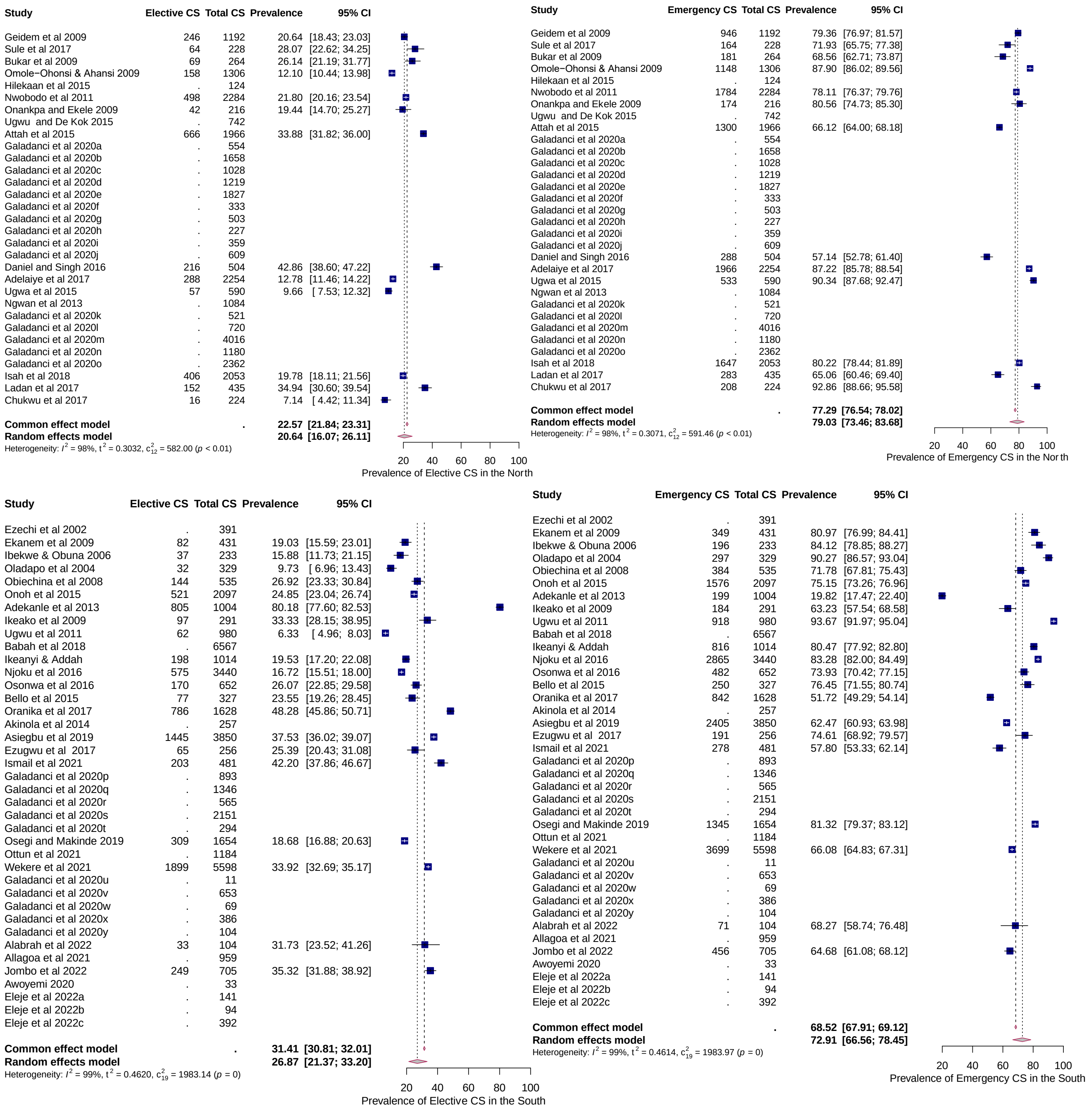

Study period: pre- and post-WHO statement

The prevalence of CS increased from 18.76% to 29.60% after the WHO statement was released. Papers with overlapping years in the middle of the forest plot

Figure S3: Forest plot of the prevalence of caesarean section prior to and after the WHO statement

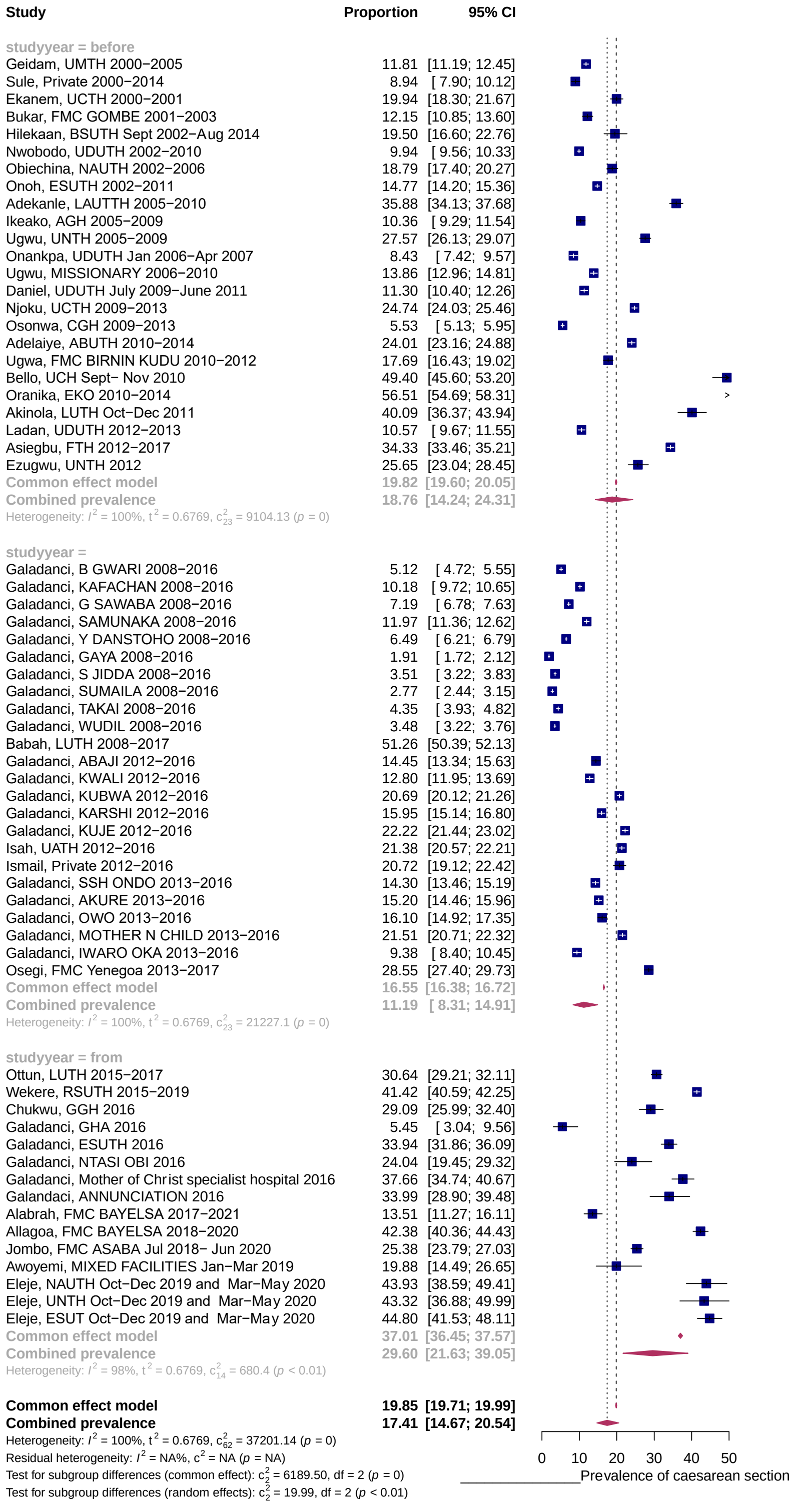

Moderator effect of study period

The study period was found to be a significant moderator of the prevalence of CS.

Figure S4: Scatter plot to show the moderating effect of study period

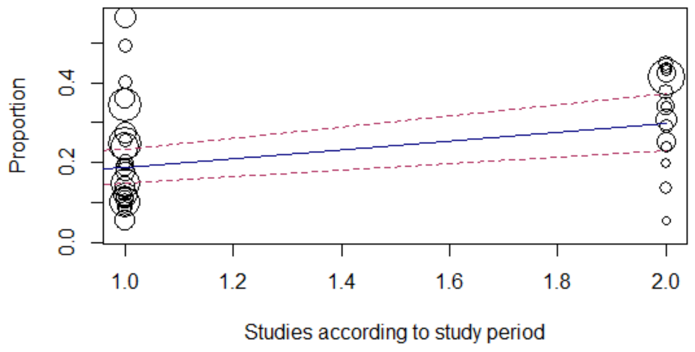

Hierarchy of facility service

Hierarchy/level of facility (secondary or tertiary) was not a significant influence on the prevalence of caesarean section.

Figure S5: Scatter plot to show the moderating effect of the class of facility service

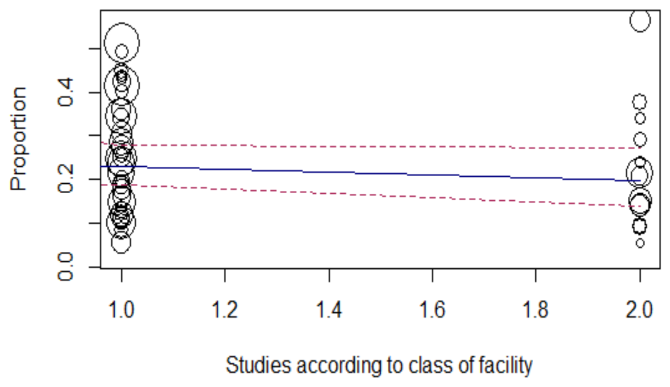

Type of facility

Facility type (public or private) was not a significant influence on the prevalence of caesarean section.

Figure S6: Scatter plot investigating the moderator effect of the type of facility

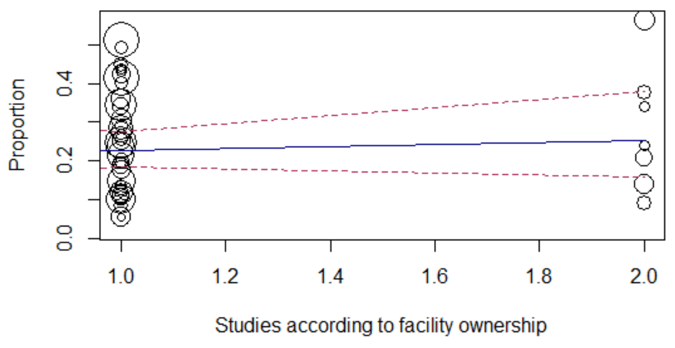

Supplement: Supplementary file 1 — Additional file 1: Table S1. Keywords for databases. Figure S1. Investigating the presence of influential studies. Figure S2. Forest plot of the prevalence of caesarean section by region and type of caesarean section. Figure S3. Forest plot of the prevalence of caesarean section prior to and after the WHO statement. Figure S4. Scatter plot to show the moderating effect of study period. Figure S5. Scatter plot to show the moderating effect of the class of facility service. Figure S6. Scatter plot investigating the moderator effect of the type of facility. [file 12978_2023_1598_MOESM1_ESM.pdf]
